# Toward personalized TGFβ inhibition for pancreatic cancer

**DOI:** 10.15252/emmm.201911414

**Published:** 2019-10-22

**Authors:** Ryan M Carr, Martin E Fernandez‐Zapico

**Affiliations:** ^1^ Schulze Center for Novel Therapeutics Division of Oncology Research Department of Oncology Mayo Clinic Rochester MN USA

**Keywords:** Cancer, Digestive System

## Abstract

Cancer can be conceptualized as arising from somatic mutations resulting in a single renegade cell escaping from the constraints of multicellularity. Thus, the era of precision medicine has led to intense focus on the cancer cell to target these mutations that result in oncogenic signaling and sustain malignancy. However, in pancreatic ductal adenocarcinoma (PDAC) there are only four abundantly common driver mutations (*KRAS*,*CDKN2A*,*TP53*, and *SMAD4*), which are not currently actionable. Thus, precision therapy for PDAC must look beyond the cancer cell. In fact, PDAC is more than a collection of renegade cells, instead representing an extensive, supportive ecosystem, having developed over several years, and consisting of numerous interactions between the cancer cells, normal mesenchymal cells, immune cells, and the dense extracellular matrix. In this issue, Huang and colleagues demonstrate how elucidation of these complex relationships within the tumor microenvironment (TME) can be exploited for therapeutic intervention in PDAC. They identify in a subset of PDAC with mutations in TGFβ signaling, that a paracrine signaling axis can be abrogated to modulate the TME and improve outcomes.

Pancreatic ductal adenocarcinoma (PDAC) is a devastatingly lethal malignancy. Despite advances in first‐line therapy, 5‐year survival has only increased from 5 to 8% over the last 30 years (Siegel *et al*, 2019). Recalcitrance is not limited to conventional chemotherapy as multiple trials employing cancer cell‐targeted treatments have failed to improve patient outcomes. Such shortcomings inspired expansion of the myopic focus on the cancer cell to a broader appreciation of the tumor microenvironment (TME) elements in PDAC pathogenesis. This shift in focus provides new opportunities for novel therapeutic intervention. The PDAC TME is composed of acellular and cellular components. Most dramatic is the characteristic desmoplastic reaction consisting of dense networks of extracellular matrix proteins such as collagen. Key cell populations include cancer‐associated fibroblasts (CAF) and a generally immunosuppressive environment including, though not limited to, regulatory tumor‐associated macrophages (TAM) and regulatory T cells (T_reg_) (Neesse *et al*, 2015). Taken together, given PDAC cells are found enveloped in a fibrotic stroma and activated fibroblasts, parallels between tumor pathogenesis and a wound‐healing response that has gone awry have been made.

The comparison of PDAC development to dysfunctional wound healing is especially striking given the key role of cytokine transforming growth factor beta (TGFβ) in both processes. In this issue of EMBO Molecular Medicine, Huang *et al* comprehensively and elegantly demonstrate a novel strategy by which TGFβ receptor 2 (TGFβR2) blockade in the stromal compartment can successfully treat PDAC tumors without intact TGFβ signaling. TGFβ effects are largely context dependent and have been shown to shape the phenotype of PDAC and its associated TME through a dual role (Massague, 2012; Ligorio *et al*, 2019). First, it activates CAFs and stimulates extracellular matrix deposition. Extent of desmoplasia has been correlated with worse patient outcomes and reduced responsiveness to chemotherapy. Second, TGFβ inhibits PDAC cell proliferation through canonical SMAD‐mediated signaling (Biffi *et al*, 2019). Therefore, while inhibiting TGFβ signaling may result in decreased CAF activation and reduced desmoplasia, it can also de‐repress PDAC proliferation. However, the Brekken group previously demonstrated therapeutic efficacy of TGFβR2 inhibition using 2G8, a murine monoclonal antibody, in a PDAC patient‐derived xenograft model so as to selectively target the stroma (Ostapoff *et al*, 2014). Huang *et al* built upon these findings to elucidate the critical TGFβ‐driven PDAC TME signaling interactions.

The extensive studies carried out primarily involved the use of not only xenograft mouse models but also two well‐established genetically engineered mouse models (GEMM) including KIC (*Kras* ^*LSL‐G12D/+*^; *Cdkn2a* ^*flox/flox*^; *Ptf1a* ^*Cre/+*^) and KPC (*Kras* ^*LSL‐G12D/+*^; *Tp53* ^*LSL‐R172H/+*^; *Ptf1a* ^*Cre/+*^). After treating these mice with 2G8, analysis of the stromal secretome identified consistently diminished IL‐6 levels. The IL‐6 cytokine has a well‐established role in promoting PDAC growth through JAK/STAT signaling (Lesina *et al*, 2011; Zhang *et al*, 2013). In fact, 2G8 treatment resulted in reduced STAT3 activation (pSTAT3) in the GEMM tumors. While PDAC cancer cells, CAFs, and immune cells are all potentially responsive to TGFβ, it was an inflammatory subtype of CAF expressing PDGFRα that was consistently found to have highest expression of TGFβR2 and IL‐6. Furthermore, it was the only cell type to secrete IL‐6 in a TGFβ‐dependent manner. Conditioned media from TGFβ‐treated CAFs was able to increase pSTAT levels in cancer cells, which was IL‐6‐dependent and abrogated when CAFs were treated with 2GB. In 3D culture, this CAF conditioned media resulted in rapid tumor growth and decreased E‐cadherin with increased N‐cadherin, markers of epithelial–mesenchymal transition (EMT). Thus, Huang *et al* defined a pro‐tumor TGFβ‐IL‐6 paracrine signaling axis between PDAC cancer cells and CAFs.

The complexity of the TME did not escape the investigators as they also interrogated the role of TGFβ and IL‐6 on the immune microenvironment. They found that 2G8 treatment of PDAC xenograft mice resulted in up‐regulation of natural killer (NK) cell activation by RNA sequencing. In fact, *in vitro* assays confirmed independent suppressive effects of TGFβ and IL‐6 on NK cells. In immune competent models, the anti‐tumor activity of the TME was significantly altered with decreased immunosuppressive cell populations such as TAMs and T_regs_ and increased stimulatory macrophages and cytotoxic T cells. This further characterized the TGFβ‐IL‐6 paracrine signaling axis as an ideal therapeutic target to not only reduce PDAC growth, but also reprogram the immunosuppressive microenvironment favoring anti‐tumor activity (Fig 1).

**Figure 1:**
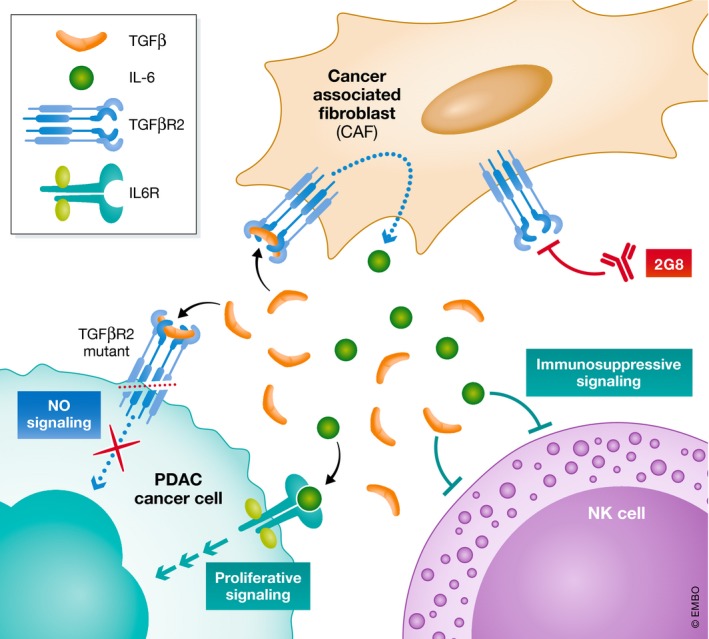
Graphical depiction of the proposed TGFβ‐IL‐6 paracrine signaling axis in PDAC TME pathogenesis TGFβ acts through its cognate receptor, TGFβR2, on cancer‐associated fibroblasts (CAF) to induce IL‐6 secretion. Both TGFβ and IL‐6 independently act on elements of the innate immune system such as natural killer (NK) cells to promote an immunosuppressive milieu. On the PDAC cancer cell, TGFβ acts through canonical signaling pathways to inhibit proliferation, while this signaling is blocked with TGFBR2 mutation. IL‐6 acts through its receptor, IL6R, to activate JAK/STAT signaling enhancing cell growth. Monoclonal antibody against TGFβR2, 2G8, disrupts this paracrine signaling network.

However, despite abrogation of TGFβ‐induced IL‐6 production by CAFs, reduced pSTAT3 in cancer cells, and reprogramming of the immune microenvironment, 2G8 treatment resulted in decreased survival. This was not entirely unexpected as inhibition of TGFβ signaling in PDAC has previously been unsuccessful (Hezel *et al*, 2012). SMAD4, a critical effector of TGFβ signaling, is one of the most commonly mutated genes in PDAC, and based on a review of The Cancer Genome Atlas (TCGA), TGFβR2 is inactivated in approximately 7% of cases (Waddell *et al*, 2015). Thus, using CRISPR‐mediated inactivation of TGFβR2, a cell line unresponsive to TGFβ was generated. In both xenograft and syngeneic murine models, the investigators remarkably found that 2G8 treatment resulted in aggressive tumor growth and tumor regression in TGFβR2 wild‐type and TGFβR2 mutant tumors, respectively.

Taken together, this extensive study from Huang *et al* illuminates key features of PDAC biology, challenges to treatment intervention, and the need for a more personalized approach. Over the course of PDAC development, a supportive niche is constructed composed of an extensive network of intercellular interactions with “normal” stromal and immune cells making up the TME. Despite this complexity, relationships between tumor cells and TME components can be exploited for successful therapeutic strategies where focusing on the cancer cell alone fails. Furthermore, considering TME dynamics allows for understanding of more complex and context‐dependent roles of “common goods” such as secreted factors like TGFβ. This study demonstrates how such mechanistic clarity of the tumor ecosystem provides insights into personalized therapeutic strategies for a deadly disease in desperate need for progress.
